# Anti-Black racism in clinical supervision: asynchronous simulated encounters facilitate reflective practice

**DOI:** 10.12688/mep.19487.1

**Published:** 2023-01-18

**Authors:** Amanda J. Calhoun, Andrés Martin, Ayodola Adigun, Shirley D. Alleyne, Kammarauche Aneni, Tara Thompson-Felix, Andrea Asnes, Marco A. de Carvalho-Filho, Laelia Benoit, Inginia Genao

**Affiliations:** 1 Child Study Center, Yale School of Medicine, New Haven, CT, 06520, USA; 2 Vagelos College of Physicians and Surgeons, Columbia University, New York, NY, 10032, USA; 3 Division of Child Psychiatry, Lakeland Regional Health Medical Center, Lakeland, FL, 33805, USA; 4 Department of Pediatrics, Yale School of Medicine, New Haven, CT, 06520, USA; 5 Department of Medical Education, University of Groningen, Groningen, 9700, The Netherlands; 6 Office of Equity, Inclusion and Belonging, Penn State College of Medicine, State College, PA, 17033, USA

**Keywords:** Racism, anti-racism, supervision, medical education, qualitative

## Abstract

Background

Racist interactions in clinical practice remain a pervasive reality for Black healthcare providers. We sought to develop a framework to inform supervisors’ actions when confronting racism in clinical practice and protecting trainees under their oversight.

Methods

We conducted a prospective study in which experienced supervisors responded to seven short, videotaped interactions between: 1) Black trainees and a simulated patient (SP) in a racist role; 2) the trainees and their respective supervisors; and 3) the trainees and their supervisors together with the SP. The clinical exchanges exemplified different types of racist *(entrenching)* or antiracist *(uprooting)* behaviors by the supervisors. After viewing each clip, participants wrote their reflections confidentially; they later joined a structured debriefing together. We used thematic analysis to identify supervisors’ behavioral patterns when confronting racist interactions.

Results

Based on the input of 52 participants recruited into five two-hour-long sessions, we categorized the behaviors of supervisors facing anti-Black racial injuries involving learners under their oversight. We organized supervisor behaviors into five interlocking domains, each with a range of possible themes: 1) *Joining*: from conciliatory to confrontational in communicating with the aggressor; 2) *Explicitness*: from avoiding to naming racism; 3) *Ownership*: from individual to shared responsibility of the event and the response to it; 4) *Involving:* from excusing to including the aggrieved party when confronting the aggressor; and 5) *Stance*: from protective to paternalistic in supporting the learner’s autonomy.

Conclusions

Our qualitative findings can provide a framework for facilitated discussion toward reflective practice among healthcare providers who may have experienced, witnessed, or intervened in anti-Black racist interactions. They can also help medical educators to inform faculty development to fight anti-Black racism in clinical practice. The video materials we developed are available for viewing and download and can be used or adapted as springboards for reflective discussion or faculty development activities.

## Introduction

Anti-Black racism in the medical workplace remains a daily reality for many. Black medical students, residents, fellows, practicing physicians, patients, and staff experience routine acts ranging from micro-aggressions and racialized dismissiveness to overt aggression ^1– 4^. Hostile conditions met by Black medical professionals contribute to disparities in recruitment, retention, and representation at all levels of practice. Racism in healthcare negatively impacts minoritized employees’ wellness and productivity and has a deleterious effect at the organizational level ^5^. Efforts to address these serious problems have not had nearly enough impact on daily practice. For example, implicit bias training, emphasizing recognition rather than communication, behaviors, or other strategies for change, does not necessarily lead to measurable changes in the workplace ^6– 8^.

In medical settings, a specific clinical challenge for supervisors is how to respond—in real-time, at the moment of charged affect—to racialized and hostile interactions involving trainees under their supervision ^9^. Supervisors responsible for the oversight, protection, and support of their racially minoritized charges are often inconsistent in their approach and have limited guidance to inform their actions. Several useful approaches to deal with microaggressions and racism in the medical workplace have been put forth ^1, 10– 12^. These studies have predominantly focused on learners rather than supervisors. The closest exception is a qualitative study in which medical students identified ideal responses by their supervisors to microaggressions ^13^.

Racist interactions are common in clinical work but are often unwitnessed. For example, it is rare for supervisors to be present at the time the trainee is on the receiving end of racialized aggression. Discussing such an event after-the-fact may remain too raw for some trainees or come too late for others. As a result of few opportunities for practice or to intervene in real time, supervisors may lack the skills or feel unprepared to combat racism in their clinical workspace. Human simulation offers a promising approach for supervisors to improve their antiracist actions in clinical practice ^14, 15^.

One way of addressing racism in clinical settings is to take a deliberately antiracist stance in education (including through simulation) and practice (including through supervision) ^16, 17^. Antiracism, which posits that racial groups are equal and supports policies that reduce racial inequity ^18^, is complementary to critical race theory (CRT) ^19, 20^. In turn, CRT considers race and racism from the perspective of power and larger historical determinants socially engrained over time. Reparative justice practices—based on the three tenets of acknowledging victims, taking concrete steps to repair harms, and validating victims as bearers of equal rights—can contribute to positive change in the learning environment through intergroup contact that emphasizes perspective-taking (e.g., between a trainee and a supervisor) ^21^.

In an effort to enhance supervisors’ concrete tools to address racist interactions in the clinical workspace, we designed and conducted a prospective empirical study to: 1) create a series of short video clips depicting anti-Black racist and antiracist clinical interactions based on the lived experience of those creating their content; 2) embed the video clips as stimuli into faculty development activities to foster reflective practice in clinical supervision; and 3) use qualitative methods informed by a theoretical context of antiracism, CRT, and reparative justice practices to identify aspects that could be leveraged toward positive change in pursuit of more equitable, supportive, and protective supervision of targeted trainees.

## Methods

Our study consisted of two distinct components: 1) creation of stimulus videos depicting racist and antiracist interactions in clinical practice; and 2) qualitative analysis of data collected during small group sessions, in which clinicians reflected on seven short videoclips.

### Ethics and consent

Participants provided written informed consent, and the Yale Institutional Review Board approved the study (protocol # 2000030453; approved April 9, 2021). Participants appearing in the videos ( *Extended data*) also provided written consent to the videos’ publication.

### Creation of stimulus videos

We created seven videos depicting the interactions and behavioral patterns outlined in Table 1. In them, trainees first interact with a racist patient; they next meet with their supervisor to discuss the case; the trainee and supervisor finally rejoin the patient.

**Table 1.** Stimulus videos of simulated encounters depicting racist and antiracist behaviors in clinical supervision.

Clip	Featured	Depicted interactions
IA	Trainee and patient in racist encounter	As a Black female physician updates a white male patient, he responds with several overt racialized statements (e.g., repeatedly questioning her credentials, implying she is a "diversity" recruit, or asking for a doctor "I can relate to.")
IIA	Trainee and supervisor in interactions that *entrench* racism and the status quo	*Frozen* trainee: a stunned reaction in which she finds it hard to say much and experiences a wilting silence. *Dismissive* racism, where the supervisor brushes off events or actions as universal ("we all have been treated this way," "it's part of the job experience"), not relevant ("it's him, not you: it's not personal"), or ignored through platitudes ("you are doing a great job, don't pay attention to that, just focus on the patient.")
IIIA		*Fleeing* trainee, attempting to negotiate out of the clinical encounter ("I'll see any other patient instead"), at times ingratiating herself through *fawning* compliments ("you are so good at dealing with complex cases.") *Benevolent* racism, where the supervisor can identify emotional hurt, perhaps even inquire whether racism has been at play. However, her response becomes oppressive in its overzealous attempt to resolve the situation prematurely.
IVA		*Forward-leaning* trainee, discussing in a direct and candid way the racist interaction she experienced– and the only example in which racism is explicitly named. *Internalized* racism, in which the supervisor identifies the racist behavior for what it is but recommends avoiding it ("it will just make things worse"), implying a role in exacerbating matters ("you mentioned racism?", "you were too direct"), and warning against possible retaliation ("he might file a complaint," "you don't want to be pegged as the ‘angry Black woman.")
IIB IIIB IVB	Trainee, supervisor, and patient in interactions that *uproot* racism and foster antiracist practice	The same three trainee-supervisor pairs now join the patient; in these interactions, supervisors demonstrate behaviors they had considered effective and supportive in their past experience: actions they felt proud of toward disrupting racist events and tense interactions; in short, ways in which they considered having been effective in the oversight and protection of their trainees. Among some of the statements by supervisors: “What you said was racist and is unacceptable”; “You are treating me very differently than her, even though she is taking excellent care of your child”; “No; we are not in agreement, and I find your comments dismissive and offensive”; “We will take care of your son, but will ask you to leave if you persist with this behavior”; “I will call security if you persist.”

*Note:* video clips have a mean duration of 5 minutes each. All videos are available for viewing or download at Figshare ^26^.

***Trainees.*** Our study is grounded in participatory action research (PAR) ^22– 24^, an approach in which an intervention's intended beneficiaries are involved in its development. In this case, those beneficiaries included five Black female physicians who had been on the receiving end of racist interactions during graduate training. Using the principles of co-constructive patient simulation ^25^, in which case scenarios and learning objectives are co-constructed by the learners rather than the instructors, they developed scripts and then demonstrated in the videos three response styles common in facing racist interactions as trainees: *freezing, fawning,* and *forward-leaning* (see Table 1)

***Supervisors.*** The supervising physicians in the videos were three senior coauthors with extensive supervisory experience. Each one represented a different demographic: a white man, a white woman, and an Afro-Caribbean woman. In anticipation of recording the video interactions, we asked each one of them to think back to supervisory responses falling into one of two categories around racism: *entrenching* (racist) or *uprooting* (antiracist). For the *entrenching* responses, supervisors recalled ways in which they or their colleagues had not addressed racism effectively, where if anything, they may have helped sustain or reify the status quo of their work environment. For their *uprooting* responses, this time demonstrating behaviors they had considered effective and supportive in the oversight and protection of their trainees.

***Simulated patient.*** A professional actor with experience working as a simulated patient (SP) followed best practices ^27^ in taking on the patient role ( *e.g.*, working with medical scripts and in medical settings, patient confidentiality, infection control, etc). All others featured in the video clips are practicing clinicians, not actors.

### Participants and study design

We recruited volunteer participants into a research session on best practices in supervision through email solicitation, using listservs from the office of Graduate Medical Education and the departments of medicine, pediatrics, and child psychiatry at Yale School of Medicine. We specifically approached, in equal parts, faculty members with at least three years of supervisory experience and senior residents or fellows with at least three years of postgraduate training. In addition, we opened recruitment to clinicians of similar seniority in other disciplines, including psychology, social work, and nursing. We informed participants that we would not collect any personally identifying information.

We conducted five sessions every other week between July and September of 2021. Each two-hour-long session consisted of four parts. In part I *(Clinical encounter,* video IA) and II *(Supervision,* videos IIA, IIIA, IVA), participants viewed short clips depicting vignettes demonstrating racist clinical interactions, as exemplified by the same supervising clinicians. Following each video, they had three minutes to enter free text comments into Qualtrics (Provo, UT) using their preferred WiFi-enabled device. Participants had the simple prompt to enter their reactions to the videos and any ideas of what they would have done differently if finding themselves in a similar situation. We conducted the study through video conferencing using Zoom (San Jose, CA); the video-clips had been pre-recorded. To prevent interaction during the first two phases, we asked participants to turn off their cameras and microphones.

After a short break, we presented a *Didactic* (III) developed with the goals of 1) enhancing education of the historical and current underpinnings of anti-Black racism in the medical system and how it fuels distrust among Black patients, 2) developing knowledge and skills to analyze racial disparities in medical practice through the lens of anti-Black racism, 3) developing the skills and tools to name racism and productively intervene in clinical settings in real time, and 4) examining antiracist attitudes and behaviors along a hypothesized continuum ^28^. To exemplify best antiracist practices in clinical supervision, we incorporated three *uprooting* (disrupting *)* video conditions into the didactic (videos IIB, IIIB, IVB). In this way, all participants got to see each of the three supervisors using both their *entrenching* (racist, II) and *uprooting* (antiracist, III) approaches in response to racist injuries. After a final *Debriefing* (IV), recorded and transcribed verbatim, participants completed their last free-text entry. The video stimuli and debriefing prompted participants to recall past experiences and reflect on what they saw and experienced during the activity. We contacted all participants by email two weeks after their session and encouraged them to provide any additional thoughts or reflections; we did not provide them with a specific prompt.

### Qualitative analysis and reflexivity

We used thematic analysis (TA) ^29, 30^ to identify supervisors’ behavioral patterns when confronting racist interactions. TA fosters flexibility in identifying underlying commonalities. Analyzing and writing occur iteratively in TA, which includes detailed accounts of the data undergirding specific themes. Aided by NVivo software (QSR International, Melbourne), two authors coded independently toward developing a joint codebook of overarching themes that reached theoretical sufficiency ^31^. Our final domains and underlying themes are supported by representative verbatim quotes.

We addressed our own reflexivity ^32^ by focusing on emotionally-laden, complex, or ambiguous subject matter, i.e., considering rather than discarding our personal and subjective views as investigators. The investigative team included physicians who had experienced or witnessed racism directly, as well as supervisors who in their own estimation had responded in ways they later deemed suboptimal. Of relevance, seven of the participants had participated in a CCPS (co-constructive patient simulation) session involving a similar case depicting racism. That session was so emotionally laden that some participants were angry and felt “put on the spot”; the SP declined to participate in that role again. An impetus for this study—and for moving it to an asynchronous video-based format—was the investigators’ goal to retain the valuable components of that learning experience without alienating or vicariously traumatizing its intended participants.

## Results

We received expressions of interest from 69 individuals, 52 (75%) of whom joined one of five sessions (median, 11 participants per session; range, 8 to 12). Volunteers were primarily physicians (n = 37, 71%), 19 of them senior trainees (37%). They self-identified as female (60%), Black (35%), and Latinx / Hispanic (13%). We intentionally oversampled Black participants: one-third in our sample, compared to the 2019 US census average of 13.4%. We identified behavioral patterns based on the qualitative analysis of participants’ contributions. We analyzed 260 free-text entries (noted with an S, for "Session," in the sections that follow), five transcribed debriefing sessions (noted with a D, for “Debriefing”), and written reflections submitted by 32 (62%) participants two or more weeks after their index session (median, three weeks; range, 2 to 7; noted with an F, for “Follow-up”). We organized behaviors around five interlocking themes, each with a range of possible approaches supervisors could resort to when confronting similar challenges (see Table 2).

### Supervisors’ responses to racist actions

***1. Joining:* from *conciliatory* to *confrontational* in communicating with the aggressor**

In response to a racist interaction, an overt approach resonated with some participants on the "step in, step up, shut (him) up" (S1) side of the spectrum, one in which "my dignity is more important than my position," (S2) and where they deemed alternatives to confrontation as unrealistic. From this perspective, absent forceful engagement with its source would risk perpetuating and tolerating similar behaviors in the future—consistent with prior experience, in which little systemic protection had taken place:

Rather than negotiating or dialoguing with patients and their families, there needs to be a boundary of absolutely no tolerance. (S3)

Others worried about a sudden and uncalled-for rush to action, advocating instead for a measured approach. By adjusting the intensity and slowing the timing of the response, they sought to balance an overriding clinical duty to the patient with the necessary support of the trainee. They advocated for behavioral scaling, concerned that forceful confrontation could exacerbate tensions and poor outcomes and defeat the building of necessary skills, such as alternative approaches through dialogue toward a middle ground of understanding:

A rush to action at the expense of reflection defies most models of leadership, which insist leaders must “get on the balcony” to see the whole picture before making a decision. How do we all become courageous instead of cowardly or rash in the face of conflict and injury is a core question here. My hope is that by pausing and going from the “arena” to the “balcony," we won’t be inadvertently deemed as racist and so foreclose dialogue, but instead move toward mutual clarification, support, and growth for all parties. (F1)

**Table 2.** Supervisors’ behavioral responses to racist acts in clinical practice.

Domain	Theme	Sample quotes
**Joining**	Conciliatory	It’s never the message; it’s always the delivery. We can say these things without our pride as supervisors competing and spiraling into a battle of ideologies. I don’t know how much is won by that. (S5)
	Confrontational	You assert your authority as a boss, a white boss, a non-Black boss—notice I said non-Black boss— that’s intentional, and you say, “We will not accept this behavior.” Period. If you can’t do this, if you can’t stop being racist, you can leave. We will take care of your child, but you cannot stay here. If necessary, we will call security. (S6)
**Explicitness**	Avoiding	Naming racism and racist behaviors always gets an emotional response. We can confront racism without the emotions unleashed by naming it, which often obscure the problem and prevent possible solutions. (S7)
	Naming	Why should I have to couch my response in terms that are "comfortable" for this aggressor? Why indeed? (S8)
**Ownership**	Individual	I want to be supportive, but I don’t want to be the rescuer, the “white savior”. How do I empower, but still be supportive at the same time? (D1)
	Shared	It is important to know what kind of support you get from the power structure in that institution because I’ve seen too many times where you will confront somebody and take a stance about their behavior, but because they’re threatened, then somebody who’s higher up comes along and tells you what you’ve done is not correct. And they validate their behavior by saying it was acceptable and yours was not. You need a supportive power structure. (D2)
**Involving**	Excusing	You don’t want to put such a toll on the person at that moment; you need to give them space and time to express what they want, with whom they want, at the moment they want. (D3)
	Incorporating	Having the discussion with the trainee in the room addressed the reality of the hostile work environment in which they work and recognized the strength it took as a person of color to speak up in this way. (S9)
**Stance**	Protective	Listen and affirm; do not direct, postulate, pontificate. (D4)
	Paternalistic	Is the pursuit of total protection from any type of harm the best goal to support trainees’ entry into attending and leadership roles? When does supervisor protection veer into protectionism akin to a parent-child relationship instead of working together as colleagues in a shared struggle to end all *isms*? (F2)

*Abbreviations:* Verbatim quotes derive from one of three study phases: S = Session; D = Debriefing; and F = Follow-up. The adjacent numbers are sequentially generated and not linked to any given session or participant.

Participants who had been on the receiving end of racialized attacks had limited trust in conciliation and efforts that they considered coddling of the aggressor rather than supporting a trainee in a subordinate position and at a disadvantage to stand up for themselves. They wanted to return to their clinical duties unencumbered and protected from hurtful acts in which there was no moral equivalence between two sides: “Nasty racist patients are not going anywhere, but neither am I.” (S4)

**2. *Explicitness*: from *avoiding* to *naming* racism**

Talking about the emotionally charged topic of racism is inherently difficult. However, labeling inappropriate behaviors and hurtful actions can be a practical entry point. Just as language shapes our understanding of the world and our actions, terminology to deconstruct racialized acts can put into words what otherwise goes unsaid—but not unfelt. Most participants found the terminology of the didactic component to be helpful: differentiating racism from racist acts or considering alternatives to euphemistic terms (e.g., *"racism*-conscious" instead of *“race-*conscious"). Many agreed that acknowledging and addressing racism starts with naming actions for what they are.

Respondents differed in their views of when best to name terms explicitly and use them for good rather than potentially weaponize them. Some conceded that naming racism may or may not be advisable with the offending party in each clinical situation but must always be addressed when supporting the aggrieved party. For example, a supervisor who does not recognize a racial injury has work ahead and further personal growth; by contrast, a supervisor who *does* become aware of such interaction but does not name it contributes to an additional injury by making their supervisee feel unacknowledged, gaslit, dismissed—or retraumatized.

Validating the experience of the supervisee is essential, including by supervisors without personal experience on the receiving end of similar disparaging comments:

How can I protect somebody I supervise from something I don't fully understand? I can support them by listening and trusting their experience, as it is their life, not mine. (D5)

Indeed, the depiction of the dismissive response was considered more hurtful and ultimately more damaging than the initial racist injury: invisibility is invalidating, it cancels out and forces shut the experience. A point of consensus was that whenever invoking racism directly, the focus must be on the person's offensive behaviors, not on the person as the offense, on *racist acts*, rather than on the person as *a racist:*

I'm not saying you are a racist, but that what you did was racist. I'm not judging you as the person you are, but your actions were racist and hurtful to the doctor I'm supervising. (D6)

There was less uniformity regarding just when and how best to name racist acts directly with the aggressor: leaving things unsaid risks sanctioning behavior, normalizing it as either typical, expected, or an understandable reaction to stress. Such elisions could entrench views not only about racism but around any of the prevailing *isms* or *othering* perceptions, including sexism, ableism, homophobia, or xenophobia.

**3. *Ownership*: from *individual* to *shared* responsibility of the event and the response to it**

Participants concurred on the need for supervisors to take a direct stance, to "own" the interaction, and to lean into their protective responsibility. Nevertheless, they often felt left to their ingenuity, intuition, or interpersonal styles. Most yearned for policies and procedures to back their actions, for even imperfect guidelines setting realistic and appropriate boundaries. Involving intermediaries such as patient relations, security services, or an anonymous reporting system were all mentioned as steps in the right direction to support injured parties not to feel dismissed or isolated.

In turn, some participants questioned what would be the optimal approach for an aggrieved trainee to take. Moreover, who would be best to share the hurtful event with. Bringing up the issue felt tiring and burdensome to some; raising it with a supervisor potentially fraught with danger, risking “being labeled unprofessional, which is just code for an individual of color who stands up for themselves." (S10) Some shared a resigned, even defeatist sentiment of "having to work in elitist institutions where this form of invalidation is a daily struggle," (S11) and where aggrieved trainees all too often come to accept generic and avoidant feedback from their supervisors. They deemed the medical enterprise a "retail business in which ‘the customer is always right,’" (F3) leaving trainees in the vulnerable position of feeling unprotected. Feeling alone when confronting experiences involving racism resulted in a sense that they could trust only certain peers or select supervisors for empathy or support, yet rarely for addressing the source grievance. Supervisors who had used their authority / positions in a constructive way to combat this type of behavior were cited as all-too-rare exemplars.

**4. *Involving:* from *excusing* to *including* the aggrieved party when confronting the aggressor**

When is it reasonable to include the racially targeted individual in addressing the encounter with its offender? In support of the inclusive approach, some participants saw a potential opportunity to model through visible actions, to have the trainee join in addressing the offense at its source. They hoped for supervisors with privilege to exemplify how addressing racism is work that should not fall entirely on those who are minoritized, as an opportunity to handle power differentials, to extend a learning opportunity to the trainee or the broader group of learners. However, the prevailing view supported a different approach: responding to the offender then and there—and at a separate time with the recipient. Several Black participants expressed doubt about not being harmed, let down, or abandoned once again, of entering a clinical space

that is never a safe environment. We might be told it is; we might like to think it is. It may be safe for some, but for those of us who are marginalized, it's not. (S12)

Other participants preferred decoupling the tasks of addressing the racist behavior with the aggressor, and processing the experience with the trainee, giving trainees time and space for reflection, gathering outside support, and gaining agency over what could be helpful as a next step. By listening first and acting only later, and by being attuned to the trainee’s needs and timing more than to their own, a supervisor could point out that support comes not only at that moment but just as importantly, between racist acts: “it’s for the long haul." (D7)

**5. *Stance*: from *protective* to *paternalistic* in supporting the learner’s autonomy**

Supervisors can become unwittingly overprotective and paternalistic in their effort to address a racist injury effectively when supporting their charges. Centering the response on the needs of their trainee is the first way to avoid that potential trap. For example, a supervisor who “just waltzes in to save the day" (S13) can convey that only they can make things better and that the aggrieved trainee is not up to the task. Moreover, the stance can become transparently performative.

Supervisors are most welcome when "validating, affirming, and witnessing, rather than when postulating or lecturing." (D8) They are protective; they inquire openly and confidentially about what is needed at the time; whenever possible embed their support within a longer-standing relationship; they realize the limitations and the risks of providing "total protection, which they really cannot promise" (D9); they are protective, but do not veer into protectionism. It is an admittedly narrow strait to navigate:

How do I empower while being supportive at the same time? I want to be there for my trainee but don't want to be their rescuer, which could feel or come across as infantilizing. (S14).

## Discussion

We consider our findings along the two parts of our study and as informed by the relevant theoretical frameworks underpinning each: 1) Short videos of simulated encounters can facilitate reflective practice; and 2) Behavioral response patterns can provide a framework for supervisors confronting anti-Black racism in routine clinical practice.

### Short videos of simulated encounters can facilitate reflective practice

Short videos permitted participants to witness and react to challenging interactions in an asynchronous way; live simulation would likely have been too raw and aversive. The videos we created can be used as either embedded or stand-alone materials to stimulate discussion about racism and antiracism in clinical supervision. We have made the clips accessible online to interested readers, but also encourage others to create videos that reflect their own experiences.

The organization of supervisor behaviors we developed is not intended as a rubric or rigid guideline; it can instead be construed as a framework derived through, and conducive to, reflective practice. Supervisors can incorporate and refine it through a three-part approach first advanced by Donald Schön in *The Reflective Practitioner* ^33^: First, by inviting reflection *in* action (i.e., *while doing;* in this case, as racist actions and responses unfold in real time). Second, through reflection *on* action (i.e., *having done;* in this case, returning to the interactions with both the source and the recipient of the racialized injury). Third, and perhaps most important, to elicit and guide reflection *for* action (i.e., *towards doing*; in this instance, to inform and revise future behaviors by reaffirming or repairing previous actions). Indeed, the CCPS model on which the source video encounters were developed is specifically designed as a structured activity to improve reflective skills in practice. Initially applied in the context of psychiatry ^34^, in this study we repurposed the same principles to examine supervisory responses to anti-Black racism.

### Behavioral response patterns can provide a framework for supervisors confronting anti-Black racism in routine clinical practice

Antiracist work is not any single group’s exclusive responsibility. Black supervisors, for example, are not inherently antiracist in their interactions, and failure to address racism effectively (as exemplified in our stimulus video of internalized racism, IVA) can cause further damage through intragroup cultural betrayal ^35^. In turn, non-Black supervisors need to commit to the iterative nature of antiracism, work that requires an enduring commitment to action and recursive learning and self-reflection: “not a state of being that, once achieved, is static and unchanging…allyship should be thought of as a verb, not a noun.” ^36^ A necessary first step in the work of antiracism is acknowledging one's own racist behaviors, embedded within a larger socio-historical context from which non-minoritized groups inherently benefit ^20^. A concrete corollary of this statement is that institutional buy-in can be critical; and yet, settings that are less welcoming or encouraging of antiracist initiatives and policies may be the ones needing them most.

We consider antiracism a lifelong journey of developmental progression rather than an ultimate point of arrival ^28^. Our understanding revolves around racist *actions*, not of *individuals* as racist: one racist action does not a racist make. Do two or three, do ten such actions? We find it more constructive to think of actions as either *entrenching* or *uprooting* racism. Discrete *actions* can be construed in the binary of racist or antiracist, as has been advanced by Ibrahim X. Kendi ^18^. And yet, those discrete behaviors do not add up to a categorical view of an *individual* as a racist or an antiracist; instead, as falling somewhere along an antiracist continuum that can be improved upon as a lifelong, perfectible goal. Consistent with that view and with the underlying tenets of PAR ^22– 24^, the ultimate use of this work will be to incorporate it into the practice of its participant-beneficiaries and fellow practitioners.

The actions of a supervisor can be considered from two interdependent angles: the need for a *response* to the situation at hand and the need for *repair* with the targeted trainee. Reparative work is essential to concretize the commitment to support and protect, lest further traumatization occur through personal or institutional omission ^37, 38^: supervisors need to be deliberate in checking in and following up. Repair can, and ideally should be extended to the community of learners so that everybody can benefit in a virtuous cycle. Beyond initiatives aimed at advancing diversity, equity, and inclusion, reparative justice practices are necessary to increase the representation of Black physicians in medicine ^39^.

### Practical implications

These video-based materials and initial framework of supervisor behaviors can help medical educators in faculty development efforts toward fighting anti-Black racism in clinical practice. The behavioral framework we derived from the responses by experienced clinicians to these asynchronous video stimuli can help supervisors engage in reflection to improve their antiracist practices in working with trainees. Healthcare providers who may have experienced, witnessed, or intervened in anti-Black racist interactions can also use the range of behavioral responses as a basis for facilitated discussion. The video materials we developed are available for viewing and download and can be used or adapted as springboards for reflective discussion or faculty development activities.

### Limitations

We recognize five main limitations. First, our stimulus videos depicted overt anti-Black racist interactions and encompassed additional elements of misogyny and xenophobia. They portrayed one extreme in the continuum of anti-Black racism—but not the most pervasive. Robin DiAngelo has described the perils of "nice racism," a more subtle and insidious form in which individuals or groups shield behind an appearance of civility that protects from the "honesty and vulnerability needed for growth and change.” ^30^ Second, we drew our sample from a single institution, reflecting circumstances unique to its environment and region's geopolitical history and current realities. Third, we are aware of the bias inherent in selecting volunteer participants ^40^. When initially approached, potential participants knew of a research study to address "clinical supervision in real time." As a result, our sample represents self-selected supervisors motivated to enhance their skills. Fourth, given that we incorporated a didactic component into the study, we can’t isolate the reaction to the videos apart from that additional content. Finally, we recognize not having analyzed findings according to sex, gender, race, or ethnicity, which could have provided additional insights into supervisors’ intersectional identities.

## Conclusions

Our work builds on the foundations by other scholars seeking effective approaches for dealing with racism in clinical settings. The behavioral dimensions we describe can be incorporated and synergize those other approaches, particularly given a commonality in theoretical underpinnings. For example, one group used a before / during / after approach for residents to confront “problematic behaviors” ^10^ organizing responses along a timeline reminiscent of reflective practice ^33^. A second group used simulated encounters to improve resident preparedness to discriminatory comments in the workplace ^15^. Two other groups ^1, 13^ have incorporated antiracism, CRT, and reparative justice into their efforts. The twelve tips from another group incorporate practical highlights that cut across the other approaches ^12^.

 Our work advances previous efforts in three main ways: 1) it is one of the few studies to derive its approach through an empirical and prospective design, and to do so using patient simulation; 2) few other studies address anti-Black racism exclusively, rather than non-specific racism or microaggressions broadly defined; and 3) to our knowledge, ours is the first study centered on supervisors, rather than on students or residents. These innovative aspects could be complementary to the works outlined above.

Notwithstanding these shortcomings, our findings can provide an approach for supervisors to engage in reflective practice, which can in turn inform their actions when next called upon to address anti-Black racism in clinical practice.

## Data Availability

Even though de-identified, we do not have permission from our institutional review board for wider distribution of the raw data transcripts of this qualitative study. The raw data supporting the conclusions of this article will be made available by the authors to *bona fide* researchers, upon reasonable request. Please contact to corresponding author ( andres.martin@yale.edu) to request access. figshare: Anti-Black racism in clinical supervision: asynchronous simulated encounters facilitate reflective practice. https://doi.org/10.6084/m9.figshare.21669506.v2 ^26^ This project contains the videos used in this research. Data are available under the terms of the Creative Commons Attribution 4.0 International license (CC-BY 4.0).
