# Supplementary material for: Anti-Black racism in clinical supervision: asynchronous simulated encounters facilitate reflective practice
Source: MedEdPublish (2016). 2023 Apr 14;13:4. Originally published 2023 Jan 18. [Version 2] doi: 10.12688/mep.19487.2 (PMC10140654; doi:10.12688/mep.19487.2)
Supplement: Peer review history

# Peer review: Reviewer response for version 2

**DOI:** 10.21956/mep.21040.r33106

**Published:** 2023-04-27

**Authors:** Javeed Sukhera

**Affiliations:** 1 Department of Psychiatry, Hartford Hospital Institute of Living, Hartford, CT, USA

Thank you for your thoughtful responses. This is a fantastic paper.

Have any limitations of the research been acknowledged?

Yes

Is the work clearly and accurately presented and does it cite the current literature?

Yes

If applicable, is the statistical analysis and its interpretation appropriate?

Not applicable

Are all the source data underlying the results available to ensure full reproducibility?

Partly

Is the study design appropriate and is the work technically sound?

Partly

Are the conclusions drawn adequately supported by the results?

Partly

Are sufficient details of methods and analysis provided to allow replication by others?

Partly

Reviewer Expertise:

Reflection, transformative learning, and equity in medical education.

I confirm that I have read this submission and believe that I have an appropriate level of expertise to confirm that it is of an acceptable scientific standard.

# Response

**Published:** 2023-04-27

**Authors:** Andres Martin

**Affiliations:** Yale University, USA

Thank you for your constructive feedback and kind words.

# Peer review: Reviewer response for version 1

**DOI:** 10.21956/mep.20880.r32900

**Published:** 2023-03-13

**Authors:** Marie-Aude Piot

**Affiliations:** 1 Necker-Enfants malade hospital, Université Paris Cité, Paris, France

This is a very interesting piece of work. This address with relevance a burning issue which needs to be defended. Authors offer useful practical tips which should be developed.

 Thank you for the opportunity to read it!

 ***

**Introduction** is streamlined and well-documented while arguing appropriately the need and originality of this study.

 ***

**Method section** is clear.

***Theoretical framework*** about racism theory as well as about thematic analysis are well set.

 The participatory research action position is really relevant in this context as theoretical framework; but this may be assumed in the whole study framework, and not only for trainees’ part. For example, debriefing (intervention) was also part of data collection (exploration). Conversely, free text comment can be considered as data collection; but – if I did not misunderstand - this can also be considered as self-reflexion fostering further reflexivity. With the third part of training, intervention and research are more intertwined (didactic influencing directly free text comment). Qualitative approach can assume this position with flexibility. But this may need to be set more explicitly reported.

 Maybe even that this should be highlighted as the originality and strength of this paper, aligned with the need of the subject: participatory action research may be an efficient way to grasp what’s going on in such unmentionable field. A neutral observatory position – as simple face-to-face interview - would miss essential part of exploration while limiting the practical implications.

 About ***creation of stimulus video***:

 Research and educational design used some of the most advanced in their area, respectively PAR and CCPS.

 Table 1 is very relevant so that readership may figure out more precisely what was played.

 Trainees and supervisors’ preparations are well-detailed.

 Actor’s training section follows SBOP’s guidelines, ensuring quality of training.

 These guideline mention that SP training needs to  “ Engage SPs in discussion and practice of role portrayal features (e.g., affect, signs and symptoms, behaviors)” and “Ensure SP readiness for the simulation activity through repeated practice and targeted feedback.”.

 Yet in this burning issue, authors may report additionally whether they had discussions with actors about their attitudes, representations and previous experiences about racism issues, or not. This may influence PS’s position unconsciously during the play, and improve educational safety conditions accordingly.

 Complementary ***data collection*** gateway are very relevant for such sensitive subject where participant may not dare tell all what they really think spontaneously; but may raised a brief limit (see limit section).

 ***

**Results** are clear, aligned with thematic analysis way of mapping data, while providing at first sight useful and practical implications.

 ***

**Discussion** provides relevant practical tips to applying results based on Schön’s approach.

 Stressing developmental and progressive approach about antiracism set this discussion is a moderate position. This elicits criticism that could be done to a militant position towards researchers.

 That said, assuming a repairing position complementary to educational missions seems essential.

 Grounding this position in repairing justice was very relevant while offering an innovating educational framework that should be followed.

 In **the limit** section, the sentence “As a result, our sample represents self-selected supervisors motivated to enhance their skills. » may be nuanced. Indeed, method section did not report motivation to participate in data collection. Other underlighting stakes could be involved and influence results; such as guilty or personal previous attitude towards black community with the aim to repair (rather than only improve), or conversely anger against antiracism with the aim to revenge, etc.

 Aligned with the limit “given that we incorporated a didactic component into the study, we can’t isolate the reaction to the videos apart from that additional content. “, the didactic component may be both the strength (of struggling against antiracism and awful reaction reported in the reflexive part) and the limit.

 On the one hand, the sensitivity of this subject and the involved educational framework may have hampered comments perceived by them as forbidden or shameful.

 On the other hand, free text comments had the opportunity to grasp spontaneous thoughts with its load of authenticity but can limit in-depth developments.

 Debriefing was a disposal which triggers reflexivity. Yet, presence of the group in the data collection could limit in-depth developments both because of pragmatic constraint or speech repartition and fear to mention unmoral speech. This may limit access to “nice racism” features (as presented by the authors in Robert DiAngelo concepts).

  ***

 I recommend the indexing of this work.

Have any limitations of the research been acknowledged?

Partly

Is the work clearly and accurately presented and does it cite the current literature?

Yes

If applicable, is the statistical analysis and its interpretation appropriate?

Not applicable

Are all the source data underlying the results available to ensure full reproducibility?

Yes

Is the study design appropriate and is the work technically sound?

Yes

Are the conclusions drawn adequately supported by the results?

Yes

Are sufficient details of methods and analysis provided to allow replication by others?

Yes

Reviewer Expertise:

Simulation-based education

I confirm that I have read this submission and believe that I have an appropriate level of expertise to confirm that it is of an acceptable scientific standard.

# Peer review: Reviewer response for version 1

**DOI:** 10.21956/mep.20880.r32937

**Published:** 2023-02-14

**Authors:** Javeed Sukhera

**Affiliations:** 1 Department of Psychiatry, Hartford Hospital Institute of Living, Hartford, CT, USA

Thank you for the opportunity to review this paper. I read it with great interest.

 The introduction is very well written and contains a concise summary of several important ideas. It could be enhanced by adding a bit more information about examples that may arise in clinical supervision that go beyond micro aggressions in a clinical learning environment. For example, supervisors themselves may experience micro aggressions from learners, and vice-versa. Also, introducing how critical reflection (as the author’s cite in the discussion) relates to their approach may be helpful. Alternatively, critical reflection could be mentioned in the methods as part of the authors’ theoretical and conceptual framework.

 The concluding paragraph of the introduction suggests that the authors conducted a prospective empirical study to create clips, embed the clips into faculty development activities, and used qualitative methods to identify aspects that could be leveraged towards positive change. This leaves the reader confused about which activities constituted the authors’ line of inquiry. Would suggest simply saying that the study sought to explore participants reactions and the videos/didactic were used as an elicitation prompt. The creation and embedding of the videos is important work but would not necessarily be considered part of the research study itself. This clarity is important to make it evident that the paper is not written around an evaluation paradigm and should be considered research rather than evaluation.

 The authors approach is grounded in participatory action research and critical qualitative epistemology. Would therefore start the section after the introduction with a section entitled, “Theoretical and Conceptual Framework” and highlight PAR, critical qualitative epistemology, and how they are complementary and suited for this study. This new section can also include the authors’ excellent 6P model which they can cite from previous work.

 The section entitled “creation of stimulus videos” could be reframed as “background and setting” with more information about how the videos were created and designed in accordance with the authors' theoretical/conceptual framework. Would therefore foreground the qualitative exploratory aspects of the study before getting to the results.

 The section on reflexivity could be enhanced by more information regarding the authors’ own experiences and how the team worked together through the analysis. For example, who was the primary coder? How were differences resolved? What was the role of the team members who were not involved in coding? The section on author contributions is helpful, however, more information would enhance the manuscript.

 Overall the results would benefit from some re-organization. The way that it is currently written with sections includes some rich description and analysis, however, the overall narrative of the results does not seem to tie together into a cohesive thread. In addition, the section titles include terms that are not explained for the reader. For example, what do terms such as “joining” or “involving” actually mean to the participants? How were they understood and perceived differently and by whom? Were the participants reacting to the videos, or the didactic, or both?

 In section 1, the third paragraph is quite fascinating and could use some elaboration about how trust or mistrust influenced participants’ reactions and perceptions.

 In section 3 on ownership, it is unclear from the current 2 paragraph how they are connected to the concept of “ownership” or what “ownership” meant to the participants. The comments on the emotional labor that participants experienced and the nature of the medical “enterprise” are important, however, the second paragraph does not seem to connect to the first.

 In the discussion, the two parts of the study are referenced. Again, it would be clearer if the study itself and the manuscript focused on the response patterns and how they may inform a framework as the paper does not contain sufficient details on video development, prototyping, etc.,

 The discussion contains some important connections with Schon’s work. It could be further enhanced if the authors could connect existing literature on critical reflection with their own 6P framework and describe how what they learned from their inquiry may provide any unique insights. The reflective model should also be introduced in the introduction and elaborated upon in the discussion, rather than the discussion alone.

 The practical implications section could us some elaboration so that medical educators may have a framework from the authors’ findings to implement/evaluate similar models in their context.

 The discussion would also benefit from the author’s perspective on areas for future research.

 Overall, an excellent example of the practical and empirical work needed to advance anti-racist praxis in medical education.

Have any limitations of the research been acknowledged?

Yes

Is the work clearly and accurately presented and does it cite the current literature?

Yes

If applicable, is the statistical analysis and its interpretation appropriate?

Not applicable

Are all the source data underlying the results available to ensure full reproducibility?

Partly

Is the study design appropriate and is the work technically sound?

Partly

Are the conclusions drawn adequately supported by the results?

Partly

Are sufficient details of methods and analysis provided to allow replication by others?

Partly

Reviewer Expertise:

Reflection, transformative learning, and equity in medical education.

I confirm that I have read this submission and believe that I have an appropriate level of expertise to confirm that it is of an acceptable scientific standard, however I have significant reservations, as outlined above.

# Response

**Published:** 2023-04-03

**Authors:** Andres Martin

**Affiliations:** Yale University, USA

**Reviewer 1**  Thank you for the opportunity to review this paper. I read it with great interest. **Thank you for your close reading and constructive recommendations.**

 The introduction is very well written and contains a concise summary of several important ideas. It could be enhanced by adding a bit more information about examples that may arise in clinical supervision that go beyond micro aggressions in a clinical learning environment. For example, supervisors themselves may experience micro aggressions from learners, and vice-versa. **Thank you. We agree, and allude to a broad range of interactions between clinical actors: Black medical students, residents, fellows, practicing physicians, patients, and staff.**  

 Also, introducing how critical reflection (as the author’s cite in the discussion) relates to their approach may be helpful. Alternatively, critical reflection could be mentioned in the methods as part of the authors’ theoretical and conceptual framework. **Thank you for this important suggestion. We have now added the following paragraph and references:** **Critical reflective practices can also support antiracist practices. By fostering an awareness of the self and the situation at hand, reflective practice can inform future actions informed by current understanding. ^21a^ Through facilitated or individual reflection, through committed reflective practice, assumptions can be revisited and new perspectives considered. ^21b^ At the core of reflective practice is the tenet that “being critically reflective is not a binary trait possessed (or not) by individuals. It is learnable through personally meaningful experiences.” ^21c^** **21a. Sandars J. The use of reflection in medical education: AMEE Guide No. 44. *Med Teach*. 2009;31(8):685-695. doi:10.1080/01421590903050374** **21b. Wilson H. Critical reflection in medical training and the biomedical world view. *Med Educ*. 2020;54(4):281-283. doi:10.1111/medu.14077** **21c. Ng SL, Mylopoulos M, Kangasjarvi E, et al. Critically reflective practice and its sources: A qualitative exploration. *Med Educ*. 2020;54(4):312-319. doi:10.1111/medu.14032**

 The concluding paragraph of the introduction suggests that the authors conducted a prospective empirical study to create clips, embed the clips into faculty development activities, and used qualitative methods to identify aspects that could be leveraged towards positive change. This leaves the reader confused about which activities constituted the authors’ line of inquiry. Would suggest simply saying that the study sought to explore participants reactions and the videos/didactic were used as an elicitation prompt. The creation and embedding of the videos is important work but would not necessarily be considered part of the research study itself. This clarity is important to make it evident that the paper is not written around an evaluation paradigm and should be considered research rather than evaluation. **Thank you. We have addressed these two points in this revised new paragraph:** **In an effort to enhance supervisors’ concrete tools to address racist interactions in the clinical workspace, we designed and conducted a qualitative research study (not a program evaluation) informed by a theoretical context of antiracism, CRT, reparative justice, and critical reflective practice to identify aspects that could be leveraged toward positive change in pursuit of more equitable, supportive, and protective supervision of targeted trainees. To that end, we first created as elicitation prompts a series of short video clips depicting anti-Black racist and antiracist clinical interactions based on the lived experience of those creating their content, which we embedded into faculty development activities to foster reflective practice in clinical supervision.**

 The authors approach is grounded in participatory action research and critical qualitative epistemology. Would therefore start the section after the introduction with a section entitled, “Theoretical and Conceptual Framework” and highlight PAR, critical qualitative epistemology, and how they are complementary and suited for this study. This new section can also include the authors’ excellent 6P model which they can cite from previous work.

 The section entitled “creation of stimulus videos” could be reframed as “background and setting” with more information about how the videos were created and designed in accordance with the authors' theoretical/conceptual framework. Would therefore foreground the qualitative exploratory aspects of the study before getting to the results.

**Thank you. We think that the previous change addresses these comments and that the distinction between the parts and their relative importance is now clear. We have opted to maintain the order unchanged, as we think it highlights the relative theoretical constructs in context of the paper’s different components. We hope this is acceptable and that you concur.**

 The section on reflexivity could be enhanced by more information regarding the authors’ own experiences and how the team worked together through the analysis. For example, who was the primary coder? How were differences resolved? What was the role of the team members who were not involved in coding? The section on author contributions is helpful, however, more information would enhance the manuscript. **Thank you. We have now added:** **All authors had an opportunity to review and comment on the iteratively developed codebook and ensuing manuscript, and helped in the resolution of coding discrepancies.**   Overall the results would benefit from some re-organization. The way that it is currently written with sections includes some rich description and analysis, however, the overall narrative of the results does not seem to tie together into a cohesive thread. In addition, the section titles include terms that are not explained for the reader. For example, what do terms such as “joining” or “involving” actually mean to the participants? **Thank you. We have added descriptive phrases to each of our five domains, realizing that they were potentially vague and abstract with them. In the case of “joining”, we now specify how the stance win question is with the aggressor, i.e., how a supervisor interacts with them:** ***Joining:*** **from *conciliatory* to *confrontational* in communicating with the aggressor**

 How were they understood and perceived differently and by whom? Were the participants reacting to the videos, or the didactic, or both? **Thank you. We note from the outset who we conducted a qualitative study in which experienced supervisors responded to seven short, videotaped interactions.**

 In section 1, the third paragraph is quite fascinating and could use some elaboration about how trust or mistrust influenced participants’ reactions and perceptions. In section 3 on ownership, it is unclear from the current 2 paragraph how they are connected to the concept of “ownership” or what “ownership” meant to the participants. The comments on the emotional labor that participants experienced and the nature of the medical “enterprise” are important, however, the second paragraph does not seem to connect to the first.

**Thank you. We now qualify ownership as “supervisors’ ownership,” which should clarify the issue.**

 In the discussion, the two parts of the study are referenced. Again, it would be clearer if the study itself and the manuscript focused on the response patterns and how they may inform a framework as the paper does not contain sufficient details on video development, prototyping, etc. **Thank you. We believe the aforementioned changes clarify this issue.**

 The discussion contains some important connections with Schon’s work. It could be further enhanced if the authors could connect existing literature on critical reflection with their own 6P framework and describe how what they learned from their inquiry may provide any unique insights. The reflective model should also be introduced in the introduction and elaborated upon in the discussion, rather than the discussion alone. **Thank you. We first mention the CCPS model in the Methods section, and include in the discussion:** **The alliterative model of reflection associated with the CCPS model proved useful, highlighting specific cognitions and actions, such as *Regulate, Relate, Reason,* *Relationships*, and opportunities for *Repair or Reaffirmation*).**  

 The practical implications section could us some elaboration so that medical educators may have a framework from the authors’ findings to implement/evaluate similar models in their context. **Thank you. We now state:** **The behavioral framework we derived from the responses by experienced clinicians to asynchronous video stimuli can help supervisors engage in reflection to improve their antiracist practices in working with trainees. Healthcare providers who may have experienced, witnessed, or intervened in anti-Black racist interactions can also use the range of behavioral responses as a basis for facilitated discussion. Our video-based materials and initial framework of supervisor behaviors can help medical educators in faculty development efforts toward fighting anti-Black racism in clinical practice.  The video materials we developed are available for viewing and download and can be used or adapted as springboards for reflective discussion or faculty development activities.**

 The discussion would also benefit from the author’s perspective on areas for future research.

**Thank you. We have added:** **Future steps could include replication and dissemination, as well as evaluation of the impact of enhanced reflective practice by supervisors on the wellbeing of their trainees.**

  Overall, an excellent example of the practical and empirical work needed to advance anti-racist praxis in medical education.

**Thank you for your kind words and for excellent feedback that has improved our manuscript.**
